# The role of expectancies and emotional load in false auditory perceptions among patients with schizophrenia spectrum disorders

**DOI:** 10.1007/s00406-019-01065-2

**Published:** 2019-09-06

**Authors:** Łukasz Gawęda, Steffen Moritz

**Affiliations:** 1 Psychopathology and Early Intervention Lab II, Department of Psychiatry, The Medical University of Warsaw, Ul. Kondratowicza 8, 03-242 Warsaw, Poland; 2 Department of Psychiatry and Psychotherapy, University Medical Center Hamburg-Eppendorf, Hamburg, Germany

**Keywords:** False perception, Perceptual abnormalities, Psychosis, Confidence

## Abstract

Cognitive models suggest that top-down and emotional processes increase false perceptions in schizophrenia spectrum disorders (SSD). However, little is still known about the interaction of these processes in false auditory perceptions. The present study aimed at investigating the specific as well as joint impacts of expectancies and emotional load on false auditory perceptions in SSD. Thirty-three patients with SSD and 33 matched healthy controls were assessed with a false perception task. Participants were asked to detect a target stimulus (a word) in a white noise background (the word was present in 60% of the cases and absent in 40%). Conditions varied in terms of the level of expectancy (1. no cue prior to the stimulus, 2. semantic priming, 3. semantic priming accompanied by a video of a man’s mouth spelling out the word). The words used were neutral or emotionally negative. Symptom severity was assessed with the Positive and Negative Syndrome Scale. Higher expectancy significantly increased the likelihood of false auditory perceptions only among the patients with SSD (the group x expectancy condition interaction was significant), which was unrelated to general cognitive performance. Emotional load had no impact on false auditory perceptions in either group. Patients made more false auditory perceptions with high confidence than controls did. False auditory perceptions were significantly correlated with the severity of positive symptoms and disorganization, but not with other dimensions. Perception in SSD seems to be susceptible to top-down processes, increasing the likelihood of high-confidence false auditory perceptions.

## Introduction

A dynamic interplay between ‘bottom-up’ (i.e. sensory input) and ‘top-down’ processes (i.e. cognitive processing, expectancies) [11, 12, 16, 25], as well as subsequent intersensory integration [17], determines human perception. Thus, perception is always a condensed and potentially distorted representation of reality. These processes may play an important role in perceptual abnormalities (e.g. speech illusions, hallucinatory-like experiences, hallucinations) in both nonclinical [51] and clinical contexts [1, 27, 62].

### Perceptual abnormalities

Perceptual abnormalities are considered as a characteristic feature of schizophrenia spectrum disorder (SSD). About 70–80% of patients with SSDs experience auditory hallucinations and various other perceptual anomalies may also be observed. In addition to hearing voices, sensory illusions, vivid images, and diminished or exaggerated auditory or visual sensations are frequently present among patients with SSD [7, 38, 47, 57].

Perceptual abnormalities are also observed among people at clinical risk for psychosis [28, 35], nonclinical individuals with frequent hallucinatory-like experiences [8], and relatives of psychotic patients [37, 64]. Importantly, perceptual abnormalities are a source of great distress [2, 5, 46] and are an important predictor of social functioning [30]. Thus, a better understanding of the mechanisms promoting these experiences may be helpful, when designing clinical interventions for patients experiencing different forms of perceptual abnormalities.

Most theoretical accounts pertaining to perceptual abnormalities in SSD aim to explain auditory hallucinations [62], i.e. a percept-like experience that occurs without adequate external stimulation. Following early models proposed by Behrendt [4] and Grossberg [22] almost two decades ago, more recent theoretical accounts assume that auditory hallucinations arise as a consequence of an imbalance between bottom-up and top-down processes [1, 31, 62]. Basic experimental studies have consistently shown that sensory input is substantially modified by top-down processes [9, 52]. For instance, prior signaling of the subsequent stimulus appearance (expectancy) facilitates recognition of the stimulus. Similarly, adequate perception is also conditioned by efficient multisensory integration (audiovisual integration is most frequently studied) [56]. A dominance of top-down processes, such as prior knowledge, expectancy, and impact of multisensory integration on perception, may prompt perceptual abnormalities [13].

### The role of top-down processes in false perceptions

Studies using variants of the signal detection task have revealed that patients with SSD tend to ascribe meaning to stimuli that are in fact meaningless (e.g. white noise or meaningless speech) [10, 18, 23, 24], that is, they tend to perceive a stimulus, when it is not present (i.e. false perceptions). For example, linguistic expectancy (e.g. presenting a semantically related word before participants have to detect the signal) increases the top-down impact on perception and results in excessive false auditory perceptions, among patients with SSD [23]. This effect is more pronounced in patients with active auditory hallucinations [60]. However, some studies suggest that although the (top–down) semantic impact on false auditory perception is exaggerated among healthy people who experience auditory hallucinations, this effect has not been confirmed in psychotic patients experiencing auditory hallucinations [15]. Interestingly, an increasing number of false auditory perceptions are related to hallucination proneness in healthy individuals [3]. Again, semantic expectancy also increases false auditory perceptions, among healthy individuals who often have hallucinatory-like experiences [59], that is, elevated perceptual anomalies. This suggests that exaggerated expectancies increase false auditory perceptions among vulnerable individuals.

### Expectancy and audiovisual integration in false perceptions

Our knowledge of the impact of top-down processing on false auditory perceptions in schizophrenia is incomplete. Most studies that have investigated the role of expectancy in false perceptions in schizophrenia [60] and hallucinatory-like experiences [59] did not control the degree of expectancy. Moreover, conclusions from the studies are often limited to semantic expectancy and do not consider the role of audiovisual integration in false perceptions. Thus, a potential impact of visual stimuli (e.g. a face) on auditory perception was not considered in studies trying to understand false perceptions in SSD better. Previous studies have consistently [36, 39] found that visual information has an important impact on auditory perception. For instance, the McGurk effect shows that if visual information (a video of a person speaking the syllable/ga/) is incongruent with a syllable presented via headphones (e.g./ba/) the participants perceive a different syllable (/da/). The McGurk effect is considered as a persuasive example of the role of multisensory integration in human perception. Although results from studies on the McGurk effect in SSD are inconclusive, other studies have shown that patients with schizophrenia tend to reveal disrupted audiovisual integration [49, 63]. However, it has not been investigated to date, whether providing rich visual stimulation (e.g. a video of a person speaking a word) increases the likelihood of false auditory perceptions, among patients with SSD.

### The present study

In the present study, we aimed to improve the understanding of the impact of top–down processes on auditory perception in SSD by creating an experimental paradigm that allowed us to control the degree of expectancy (low, intermediate, and high). We manipulated semantic and visual expectancies in different conditions, which allowed us to investigate, whether visually congruent stimuli (a visual presentation of a mouth spelling the presented word) in addition to semantic expectancy (a word clue before the participant needs to recognize a word) would increase false perceptions more than semantic expectancy alone would. We hypothesized that false auditory perceptions would increase with the degree of expectancy. We expected that this effect would be exaggerated among patients with SSD, as compared to healthy controls. Furthermore, we extended our interest on the impact of top-down processes by also investigating the role of the emotional content of stimuli based on cognitive models [12]. Emotional processes play an important role in false perceptions, including hallucinations [2]. Some studies have found that negative emotions precede the onset of auditory hallucinations [8]. In their cognitive model, Waters et al. [12] integrated the findings from studies on the role of emotional processing with the knowledge on the cognitive mechanisms of false-perceptions, particularly auditory hallucination. The authors suggested that emotional processes form the vulnerability to psychosis and significantly affect cognitive processes related to false perceptions. More specifically, it is hypothesized that false perceptions arise as a consequence of an interaction between hypersalient stimuli (e.g., emotionally meaningful for the individual) and top-down processes [12]. Nevertheless, the interaction between cognitive and emotional processes and false perception in schizophrenia spectrum disorders remains understudied. Hence, according to the described model, in the present study, we hypothesized that a negative emotional load will exaggerate false perceptions. Finally, we explored the relationship between symptom severity and false auditory perceptions in the clinical group.

## Methods

### Participants

#### Schizophrenia spectrum disorders (SSD) group

Thirty-three in- and outpatients diagnosed with SSD (females *n *= 18 and males *n *= 15) took part in the study after informed consent had been obtained. Patients were recruited from the Department of Psychiatry and Psychotherapy, University Medical Centre, Hamburg-Eppendorf, Germany. All patients fulfilled schizophrenia (*n *= 28) or schizoaffective disorder (*n *= 5) diagnostic criteria according to the *DSM*-*5*, following a clinical interview with the Mini International Neuropsychiatric Interview (MINI 5.0). They were clinically stable (i.e. exhibited no agitation, aggressive behavior, or acute symptoms) and were on a stable dosage of antipsychotic medication. Patients with an equivocal diagnosis, current alcohol or any other substance dependence, or a severe neurological disease were excluded from the study.

#### Healthy control group

The healthy comparison group also consisted of 33 participants (females *n *= 15 and males *n *= 18), matched to the clinical group by gender, age, and education. All healthy participants were recruited by word of mouth and were screened for the presence of psychiatric disorders, again using the MINI 5.0. Participants with any history of neurological or psychiatric disorders were excluded from participation. The local ethical committee approved this study.

#### Psychopathology

All patients were assessed with the Positive and Negative Syndrome Scale (PANSS) [29] following a structured clinical interview for schizophrenia symptom severity. The PANSS is considered the gold standard for the assessment of symptom severity in SSD. We did our calculations using the following syndrome-based five-factor solution of PANSS [58]: positive symptoms, negative symptoms, disorganized symptoms, excitement, and emotional distress.

#### False perception task

For the purpose of our study, we developed a new false perception task. Some of the existing false perception tasks focus on one type of expectancy [3], mainly semantic [10, 14]; we aimed to design a task allowing for manipulation of expectancy to investigate whether expectancies affect true and false auditory perceptions. The false perception task contained three conditions for expectancy and two different conditions for emotional content, neutral and negative valence. In the ‘low expectancy’ condition, the content of the target stimulus was not introduced by any clue. In the ‘intermediate expectancy’ condition, the content of the target stimulus was presented (the same word as the target stimulus) before the target stimulus was shown. In the final condition, ‘high expectancy’, participants were presented with an integrated and congruent audio and visual stimulus of an actor spelling out the word. Participants had to decide (press a button) whether or not they had heard the word and how confident they were about their decision (sure vs. unsure). The instructions clarified that some words would be heard clearly, but others would be heard just on the threshold of normal human hearing. In the instruction, it was emphasized that participants’ task is only to react whether the stimuli is heard or not and not whether it is presented on the screen (i.e., ‘intermediate’ and ‘high expectancy’ conditions). In fact, 60% (*n *= 108) of all stimuli were present and audible and 40% (*n *= 72) were not audible. All stimuli were presented across the background of nonverbal ‘street noise’ through stereo headphones.

**Fig. 1:**
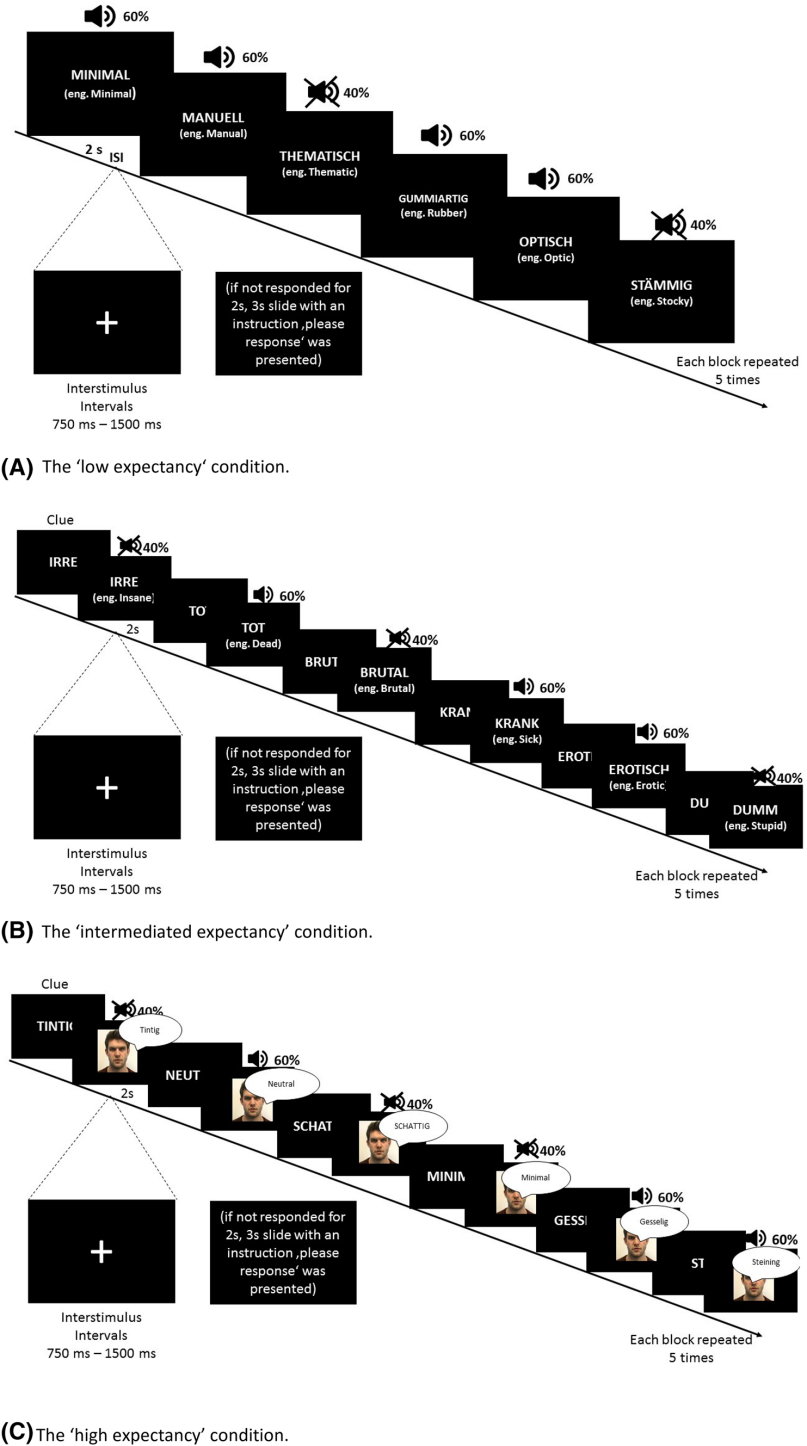
The design of a false perception task. False perception task consists of three main conditions: **a**–c, which varies on the degree and the type of the expectancy. In addition, all conditions consist of neutral and emotional words. Some of the words were audible (60%—auditory stimuli via headphones) and some of them were not audible (40%). All stimuli were presented with the street noise background. Participants had to response whether they hear or not a word. They were also asked whether they are sure or unsure in their response. False auditory perceptions were calculated as incorrect responses of heard word, when in fact no auditory stimuli were provided. In the condition A, the content of the target stimulus was not introduced by any clue. In the condition B, the content of the target stimulus was presented (the same word as the target stimulus) before the target stimulus was shown. In the final condition **c**, participants were presented with an integrated and congruent audio and visual stimulus of an actor spelling out the word

The stimuli consisted of 30 emotionally negative and 30 emotionally neutral words from the Berlin Affective Word List Reloaded [61]. Stimuli were grouped into blocks of six words. Six different blocks (2 emotional loads of the words × 3 conditions) were repeated five times. Stimuli within the block were pseudorandomized and the order of presentation of the blocks was randomized to the participant. Each word was repeated three times in a different condition (expectancy), resulting in a total number of 180 trials. Each target stimulus was present for 2 s, and then, if the participant did not respond, they were given an additional 3 s to respond after the stimulus had occurred. Inter-stimulus intervals were set up in a range between 750 and 1500 ms. Between blocks, intervals ranged between 1500 and 2500 ms. Stimuli were presented using SuperLab 5.0 software (see Fig. 1 for an overview of the false perception task design).

#### Data analysis

The number of correctly recognized audible words and the number of incorrectly recognized words (recognized, when in fact no audio stimuli were presented) were calculated for all conditions. Unreliable responses (reaction time below 500 ms) were removed from the analyses blind to results. Hence, the main analysis was performed based on the number of correct recognitions of heard words and incorrect recognitions of words that were not presented.

A four-way 2 × 3 × 2 × 2 ANOVA was calculated with group (SSD vs. control) as the between-subject factor and expectancy (‘low expectancy’, ‘intermediate expectancy’, ‘high expectancy’), emotion (neutral words vs. negative words), and confidence response (‘sure’ vs. ‘unsure’) as the within-subject factors. Hence, the main effect of group and the main effects of different conditions were considered along with interaction effects. We analyzed hits (i.e. audible words that were recognized as heard) and false perceptions (i.e. words not presented that were recognized as heard) separately. We predicted that the higher the expectancy, the higher the number of false perceptions would be. We predicted that this pattern would be exaggerated among patients with schizophrenia as evidenced by a significant interaction between the type of condition and group for the false perceptions response. We also performed analyses of the impact of the false perception task on true perceptions (i.e. hits). We expected that hits would be facilitated by stronger expectancy.

Based on prior studies, our analysis was followed by an ANCOVA accounting for general performance (correctly recognized heard words) to ensure that the outcome was not determined by overall accuracy per se.

Correlational analyses were performed to check the relationship between false auditory perceptions and psychopathology (scores on the PANSS) within the clinical group. Total scores of the PANSS as well as all of its five dimensions were correlated with auditory false perceptions. In addition, we performed a separate correlational analysis to investigate the relationship between delusions (P1 item) and hallucinations (P3 item) and false perceptions.

## Results

### Group characteristics

The groups did not differ with regard to gender, sex, or years of formal education. Fifteen patients had had active delusions (PANSS P1 item above 2) and eight patients had had active hallucinations (PANSS P3 item above 2) within one week prior to the study. Participant characteristics are presented in Table 1.

**Table 1:** Demographic and clinical characteristics of groups

	Schizophrenia spectrum disorder group (*n *= 33)	Healthy controls (*n *= 33)	Statistics
Gender [male/female]	13/20	18/15	*X*_(1)_^2^ = 1.521, *p *> 0.2
Age	35.82 (11.22)	41.33 (14.80)	*t*_(64)_ = 1.706, *p *= 0.09
Years of formal education	12.14 (1.50)	11.96 (1.31)	*t*_(64) = _0.480, *p *> 0.6
Duration of psychosis	7.11 (9.48)	–	
*PANSS*			
P3 item (hallucinations)	1.66 (1.19)	–	
P1 item (delusions)	2.39 (1.76)		
Positive	12.00 (4.90)	–	
Negative	13.00 (5.22)	–	
Disorganized	13.18 (4.01)	–	
Excitement	10.57 (2.15)	–	
Emotional distress	14.27 (4.27)	–	
PANSS total rating	46.81 (12.27)	–	

### The impact of expectancy on the detection of an auditory signal (hits)

In the first step, we checked the impact of different conditions on the correct recognition of presented words (hits). The main effect of group was significant, *F*_(1, 64_) = 4.568, *p *= 0.036, _partial_ *η*^2^ = 0.07, suggesting that the SSD group had significantly fewer hits. Similarly, we found a significant main effect of expectancy, *F*_(2,63_) = 24.266, *p *< 0.001, _partial_ *η*^2^ = 0.435; paired *t* tests suggested that both groups had more hits in the high expectancy condition compared to immediate or low expectancy. This suggests that both groups benefited from manipulation of expectancy by having more hits for conditions with a higher level of expectancy. Please note that at the same time, groups did not differ on the number of misses (no response provided), *F*_(1,64)_ = 1.36, *p* > 0.25, _partial_ *η*^2^ = 0.02. Furthermore, we found main effects of emotions, *F*_(1,63)_ = 35.056, *p *< 0.001, _partial_ *η*^2^ = 0.354, and confidence, *F*_(1,64)_ = 93.885, *p* < 0.001, *η*^2^ = 0.595, on hits. Post hoc paired t tests revealed that controls, *t*_(32)_ = 5.565, *p* < 0.001, as well as the SSD group, *t*_(32)_ = 2.929, *p* < 0.01, had more hits for emotionally negative words. Controls, *t*_(32)_ = 7.59, *p* < 0.001, and the SSD group, *t*_(32)_ = 6.18, *p* < 0.001, had significantly more hits with higher confidence (‘I am sure’). The expectancy × group interaction was nonsignificant, *F*_(2,63)_ = 2.713, *p *= 0.074, _partial_ *η*^2^ = 0.08, suggesting an equal impact of conditions on hits in the two groups. The emotion × confidence × group interaction was significant, *F*_(2, 63)_ = 5.654, *p *= 0.02, _partial_ *η*^2^ = 0.08. This result emerged, because patients made more hits with a high degree of confidence, when emotionally negative words were provided. Other effects were not significant.

### The impact of expectancy on false auditory perceptions

Our analysis revealed a significant effect of group, *F*_(1,64)_ = 5.848, *p *= 0.018, _partial_ *η*^2^ = 0.08. At the same time, the main effect of condition was significant, *F*_(2,63)_ = 6.731, *p *= 0.002, *η*^*2*^ = 0.18, whereas the main effects of emotion and confidence were insignificant (*p *> 0.5 and *p *> 0.3 respectively). There was an interaction between confidence and group at the border of statistical significance, *F*_(2,63)_ = 3.94, *p *= 0.05, _partial_ *η*^2^ = 0.06, suggesting that the SSD group tends to commit more false recognitions with higher degree of confidence.

**Fig. 2:**
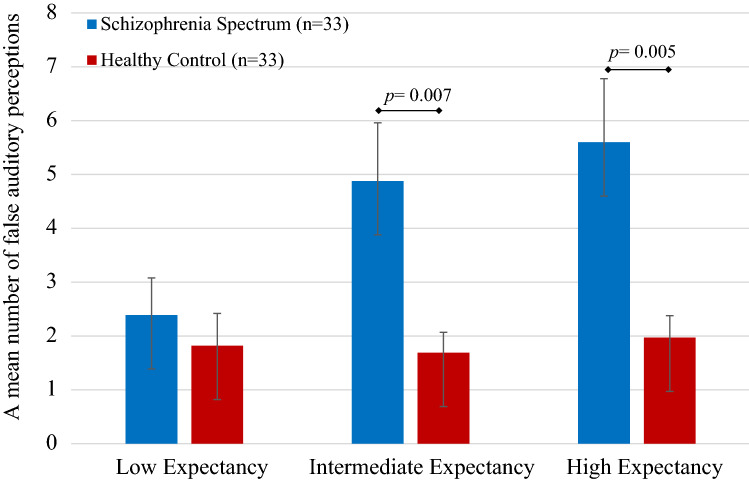
Group differences in auditory false perceptions

The hypothesized effect of the interaction between expectancy and group was significant, *F*_(2,63)_ = 5.224, *p *= 0.008, _partial_ *η*^2^ = 0.14. Post hoc t tests revealed that SSD group committed significantly more false perceptions from controls in the high expectancy, *t*_(64)_ = 2.905, *p* = 0.005, and in the intermediate expectancy condition, *t*_(64)_ = 2.77, *p *= 0.007, but not in the low expectancy condition, *t*_(64)_ = 0.627, *p *= 0.54. This effect remained significant even after controlling the general performance, *F*_(1,63)_ = 5.836, *p *= 0.019, _partial_ *η*^2^ = 0.09. Group differences in the total number of false auditory perceptions are presented in Fig. 2.

Additional analyses were performed to test the differences between expectancy conditions in the two groups separately. The results suggested that patients committed significantly more false recognitions in the high expectancy condition compared to the low expectancy condition, *t*_(32)_ = 4.336, *p *= 0.001, and at the level of a statistical trend for the intermediate expectancy condition, *t*_(32)_ = 1.952, *p *= 0.06. Patients had more false perceptions in the intermediate expectancy condition than in the low expectancy condition, *t*_(32)_ = 3.996, *p *< 0.001. Contrary to the clinical group, as suggested by the paired *t* tests, the healthy controls group had the same number of false auditory perceptions across all expectancy conditions.

### The relationship between false auditory perceptions and symptom severity

Correlations within the clinical group revealed that top-down errors were significantly related to positive symptom severity, *r *= 0.43, *p *= 0.01, and disorganization, *r *= 0.37, *p *= 0.03, as well as to total score on the PANSS, *r *= 0.38, *p *= 0.03. An additional correlation analysis of the relationship between hallucinations, delusions, and auditory hallucinations revealed that top-down errors were significantly related to delusions, *r *= 0.41, *p *= 0.02, and to hallucinations at a statistical trend, *r *= 0.31, *p *= 0.09. False auditory perceptions did not correlate with age or years of education in either group.

## Discussion

This study investigated the role of expectancy in false auditory perceptions in schizophrenia spectrum disorders (SSD). As hypothesized, our results showed that false auditory perceptions increased along with expectancy in the SSD group only, suggesting an exaggerated impact of top-down processes on perception in this group. As expected based on prior studies [15, 48, 59], higher semantic expectancy as well as rich visually congruent information, significantly facilitated the recognition of the auditory stimuli in both groups. Results suggest that both groups benefited from top–down modulation that facilitated their recognition of the auditory stimuli in the white noise background, which is in line with studies showing a preserved priming effect (top–down processing), when patients with SSD make perceptual [53] or other decisions [50]. Contrary to our expectation, we did not find any effect of emotions on false perceptions, neither in the schizophrenia spectrum group nor in the healthy controls.

We hypothesized that when the expectancy was increased, the number of false auditory perceptions would increase in the clinical group. Results of the impact of expectancy on false auditory perceptions are in line with this hypothesis. First, we found that the clinical group had significantly more false auditory perceptions, that is, SSD patients more often recognized an auditory stimulus, when in fact it was not present. Second, in line with our predictions, expectancy impacted false auditory perceptions only in the clinical group. Notably, when no expectancy was induced, both groups had a similarly low number of false auditory perceptions. As anticipated, with higher expectancy the number of false auditory perceptions linearly increased, but only in the clinical group. Group dissociation in these two conditions tentatively suggests an exaggerated impact of top-down processes on false auditory perceptions, among SSD patients. In particular, a combination of semantic expectancy and congruent rich visual information resulted in the highest number of false auditory perceptions in the white noise, among SSD patients. Importantly, our results showed that a higher number of false auditory perceptions were predominantly related to psychotic symptoms and disorganization, which suggests that a dysfunctional top-down modulation of perception may be associated with the severe reality distortions that are one of the core characteristics of SSD. Contrary to our second hypothesis and models suggesting an interplay between emotional and cognitive processes in false perceptions [62], we did not observe an interaction between expectancy and emotional load of the stimuli on false perceptions in both groups.

### Comparisons to previous studies

Our results are in line with a limited number of studies showing a stronger impact of top-down processing on auditory perceptions in patients with SSD [60] and healthy individuals with psychotic-like experiences [3], including those who hear voices [14]. We extended previous studies by showing that not only does semantic expectancy increase false perceptions in white noise, but additional rich visual information substantially further increases the risk of incorrect perceptual decisions. Furthermore, we found that exaggerated top-down regulation leading to false auditory perceptions was associated with a tendency to report higher confidence. As a consequence, SSD patients made not only more false perceptual decisions under the higher top–down influence, but also, likely due to ineffective metacognitive processes, their false decisions were determined with a higher degree of confidence, which might disturb adequate regulation of behavior. Indeed, a consistent body of studies has provided results showing deficits in metacognitive awareness, particularly overconfidence in errors associated with different decision-making tasks in SSD [20, 32]. In their two-stage cognitive theory of positive symptoms, Moritz and colleagues [42] summarized this consistent body of studies and suggested that an interaction between dysfunctional metacognition (e.g. inadequate confidence) leads to a lower decision threshold for a hypothesis that further shapes both delusions and hallucinations. Future studies that combine false perceptions and confidence are required to provide more data on the relationship between metacognitive processes and perception in SSD. Our second hypothesis stating that negative emotions may further impact false perception via an interaction with expectancy was negatively verified in this study. However, whether this finding contradicts recent cognitive accounts of hallucinations suggesting an important role of the interaction between emotional and top-down processes [12] deserves further careful investigation in future studies. In the present study, we manipulated the emotional load of the stimuli (words) by providing a contrast between neutral words and words with emotionally negative content. Hence, our manipulation might not cause significant emotional change, because even the differences between two types of word in terms of emotional load were significant, the words might not be personally meaningful to the participants. For instance, recent study conducted among patients with dementia with Lewy bodies, which often presents with psychotic symptoms, has shown significant impact that inducing negative mood significantly increases false perceptions [11]. Unfortunately, in our study, we did not check if our manipulation successfully induced negative emotions in the participants, which may be an important shortcoming that may be addressed in future studies.

### The theoretical and clinical meaning of the findings

Although different cognitive accounts of hallucinations [62] vary in the details or in the emphasis that is assigned to various cognitive factors, most apply elements from predictive coding models [26, 54] and share the opinion that perception is an active process involving a dynamic inference modulated by prior knowledge (e.g. beliefs, expectancies). Recently, in a theoretical paper, Corlett and colleagues [13] stress the role of prior expectancies (e.g. beliefs), especially the so-called ‘strong priors’, as ‘*a critical elicitor of hallucinations’* (p. 114). Our results confirm that top-down processes have an exaggerated impact on perception in this group by increasing the tendency to overperceptualize (i.e. have false perceptions). Top-down processes were found to differently affect true and false perceptions in the healthy controls and the SSD group. Prior studies found that expectancy in auditory perceptions modulates the activity of frontal and temporal brain regions in healthy individuals [43]. In individuals with SSD, an exaggerated impact of priors on false perception may be a consequence of the inadequate activation of frontal and temporal regions and anomalies in their connectivity that is observed in SSD [34] and may underlie deficits in multisensory integration in schizophrenia [56]. As previous studies have suggested, individuals prone to hallucinations have stronger beliefs that priors (e.g. expectancies or knowledge) are accurate and they are less likely to update their knowledge or expectancies when confronted with new evidence [45]. It is likely that these beliefs (strong priors) may increase decision-making confidence in patients and lead to perception overregulation in the frontal regions. Further functional neuroimaging studies of false perception are required to provide empirical evidence of this hypothesis.

Our results may have important clinical implications. Given the fact that hallucinations often co-exist with delusional beliefs, the latter treated as strong priors [13] may considerably modify patients’ perceptions. Importantly, non-delusional beliefs also precede hallucinations [55], and thus may form another set of potential priors modulating perception. Further research on cognitive priors and their interaction with perception in SSD is needed. Furthermore, a tendency to overperceptualize may underlie the deficits in discriminating between fantasy and reality (i.e. self-monitoring) that may influence severe reality distortions in SSD [19, 21]. Hence, linking existing clinical interventions, targeting cognitive biases to ameliorate delusions as well as other dysfunctional beliefs [40, 41], and developing new techniques to work directly with perceptual anomalies may be beneficial in developing new therapeutic protocols for patients. Addressing the impact of strong priors, i.e. expectancies, on perception may be an important aspect of cognitive interventions for perceptual abnormalities. Psychoeducation that translates ideas of predictive coding into everyday experience (e.g. hearing a phone ringing, when expecting an important message, when in fact no one is calling) may be useful during the first stage of working with patients experiencing perceptual abnormalities, in particular hallucinations and may foster patients’ understanding of their distressing experiences. A controlled exposition on false auditory perceptions and a demonstration of the impact of expectancy on perception during therapy sessions might provide a behavioral exercise that, when combined with a discussion linking the experience with perceptual anomalies during psychotic episodes, could be potentially useful for patients.

### Limitations

Although we tried to develop an objective measure of false perception, we cannot rule out the impact of suggestibility, which has been observed in some studies. Expectancy and suggestibility are somehow linked. Further studies may use neuroimaging techniques (e.g. EEG recording) to validate participants’ false perception responses with activation of relevant primary cortices (e.g. activation of the temporal cortex for auditory stimulus). Our primary aim was to investigate the impact of expectancy on false perception in SSD, and thus we did not split our sample according to their psychopathological status. We recruited stabilized patients with only a minority who experienced hallucinations a week prior to the assessment. As a consequence, although our results demonstrate a tendency of SSD patients to exaggerate false perception under a high expectancy condition, our study was underpowered to elucidate whether elicited false perceptions are related to the clinical symptom profile (e.g., active hallucinations). The results of our correlational analysis should be thus considered as preliminary and should be verified on a larger sample, including participants with active psychotic symptoms. Moreover, future studies may benefit from investigating also the relationship between hallucination proneness, expressed as a trait-marker and false perceptions [59]. For this purpose, scales assessing hallucination proneness may be useful (e.g., Launay-Slade Hallucination Scale [33]). It should be also noted that the linkage between false perception and psychopathological phenotype should not be limited to hallucinations. Although preliminary, our results suggested also some linkages with disorganization and delusions. In the SSD, a wide range of different perceptual anomalies are observed and hallucinations represent only one feature among these. Future studies may address this by combining experimental tasks, measuring false perceptions with enriched analysis, and investigation of patients’ experiences. As in our study, most of the research uses PANSS. Utilizing other scales, assessing perceptual anomalies more broadly [6, 44] may provide some additional insights into the field. More in-depth investigation of the impact of expectancy on false perceptions and its relation to psychopathological features will require investigation with a larger sample.
